# The influence of foreign language and a word-color unitization strategy on the age-related associative deficit

**DOI:** 10.3389/fpsyg.2026.1824417

**Published:** 2026-07-28

**Authors:** Ricarda Endemann, Erika Borella, Elena Carbone, Enrico Sella, Siri-Maria Kamp

**Affiliations:** 1 Neurocognitive Psychology Unit, Trier University, Trier, Germany; 2 Department of General Psychology, University of Padua, Padua, Italy; 3 IRCCS San Camillo Hospital, Venice, Italy

**Keywords:** aging, associative memory, foreign language, strategy use, unitization

## Abstract

**Introduction:**

Older adults typically show a disproportionate decline in associative, relative to item, memory. Here, we investigated the role of stimulus presentation in a foreign language, as well as the impact of a unitization instruction for binding word-color pairs into a single unit, in this effect.

**Methods:**

Italian younger (*n* = 34) and healthy older (*n* = 17) adults completed a word–color associative recognition memory task in their native language and in German (foreign language) under two inten¬tional encoding conditions: Without strategy instruction and with an instruction to generate a mental image of the object in the associated color (unitization strategy).

**Results:**

The group of older adults, which overall reported high levels on cog¬nitive reserve (CR) proxies, showed no evidence of a relative associative deficit in their native language without strategy instruction. However, in the foreign language and with the unitization strategy instruction, an age-related associative deficit in older adults emerged. Hence, the older adults may have been able to compensate for associative binding challenges when task demands were rela¬tively low, but not when demands increased. When no strategy was instructed, there was a tendency for a stronger relative associative memory deficit in older, compared to young adults in the foreign language. However, this pattern disap¬peared in a comparison where young adults were matched to older adults in foreign language proficiency, and hence there was little evidence for a modu¬lation of the relative age-related associative memory deficit in older adults by language. A higher increase in associative memory performance was, then, found from the object-color unitization strategy for young than for older adults, effec¬tively increasing the age-related associative memory deficit. Across age groups, memory performance increased more by strategy use in the native than in the foreign language.

**Discussion:**

Together, these results improve our understanding of the con¬tribution of foreign language and unitization strategy instructions, to age-related associative memory decline.

## 1 Introduction

Episodic memory exhibits marked age-related declines in the ability to encode, consolidate, or retrieve multiple items as part of cognitive aging. Specifically, while older adults often demonstrate comparable performance to that of younger adults in tasks that test memory for individual information units (item memory), they typically exhibit pronounced deficiencies in associative or source memory (Naveh-Benjamin, 2000; Old and Naveh-Benjamin, 2008). Although this pattern has been well-replicated (Old and Naveh-Benjamin, 2008), to date the mechanisms behind the effect are unclear. To better understand these mechanisms, the present study tested the idea that an enhanced effort to process item features, experimentally manipulated by presenting words in participants’ native vs. a foreign language, contributes to older adults’ reduced associative memory. Further, we examined the benefit of an effective encoding strategy on young and older adults’ item and associative memory in both their native and the foreign language.

### 1.1 Item processing and associative memory

In young adults, stimuli that are visually degraded or difficult to identify can lead to associative deficits that resemble the associative deficit in older adults (Naveh-Benjamin and Kilb, 2014). Further, a trade-off between encoding item features, at the expense of encoding connections between different items, was shown through experimental manipulations by Forester and Kamp (2023). As sensory processing is subject to age-related decline with aging, visual and auditory signals may be encoded and processed in a more error-prone way in older adults and perceptual processing of individual items becomes more effortful (Monge & Madden, 2016; Thiel et al., 2016). The encoding of feature-rich stimuli thus necessitates a greater allocation of cognitive resources, but the required resources are limited in aging (Chen and Naveh-Benjamin, 2012; Zacks et al., 1987). This, in turn, could result in an enhanced item processing focus, at the expense of lowered associative processing in older adults. Supporting this idea, Kamp et al. (2022) found event-related potential (ERP) subsequent memory effects (SME) in older adults during picture pair encoding, which reflect specific encoding mechanisms indicative of perceptual processing rather than elaborative, inter-item processing. In young adults, by contrast, a frontal slow wave SME indicated inter-item elaboration.

Another way to test the role of item processing in the associative deficit is via a manipulation of visual stimulus complexity. If an item encoding focus, at the expense of associative encoding, emerges in older adults due to difficulties with basic, perceptual processes, the age-related associative memory deficit in older adults should be increased when stimuli are visually complex and rich in detail. To test this, Bender et al. (2017) had participants study face-name combinations containing either black-and-white face images without background, or color images of faces that showed variable features and a background. They found that associative memory for the face-name association was disproportionally reduced for the visually more complex face images, supporting the hypothesis developed above. However, the more complex images contained not only more item features, but also additional contextual information, preventing a clear interpretation regarding an item focus at the expense of associative encoding. Endemann and Kamp (2024) presented pairs of object images with no background either as colored photos (high complexity) or as simpler, schematic black and white cliparts (low complexity). While ERPs during recognition in young adults showed that more complex images led to more effortful slow item recollection processes, both age groups showed increased item and increased associative memory for the complex image pairs. There was hence no evidence for the hypothesized trade-off between item complexity and associative memory, and the age-related associative memory deficit was unaffected by item complexity. However, one limitation is that semantically meaningful and familiar objects were used, in which case further visual details could support semantic processing, which could counteract the putative trade-off between item and associative processing.

Other evidence indicates that semantic meaningfulness and familiarity of stimuli modulate age-related associative memory deficits. Older adults generally perform comparably to younger adults on item memory for meaningful lexical items (words), but their performance declines markedly for pairs including meaningless nonwords (Naveh-Benjamin, 2000), which presumably require more processing effort. Further, Shing et al. (2008) reported that associative memory for word pairs was particularly challenging in older adults when one word was presented in an unknown foreign language, and despite undergoing extensive training in memory strategies, older adults demonstrated minimal improvement in associative memory for these pairs.

To manipulate item processing effort, in the present study we presented verbal stimuli either in participants’ native language (Italian) or in a foreign language in which they had only basic proficiency. Due to lower familiarity and weaker phonological sensitivity (sound-based intuition), presenting words in a foreign language should result in higher item processing effort (Bubbico et al., 2019), which in turn may reduce associative memory (Forester and Kamp, 2023). As older adults would be more strongly affected by this increase in item processing demands, we expected an enhanced associative memory deficit in the foreign language. An alternative possibility can be derived from Francis et al. (2018). They demonstrated that language-related familiarity—encompassing language experience, proficiency, and exposure (e.g., to pronunciation and orthography)—can influence individuals to rely on surface-level cues rather than on recollection of specific prior encounters, thereby increasing susceptibility to item recognition errors. In contrast, source memory appears to be grounded in conceptual-level representations unaffected by language familiarity. Taken together, the present study examined how presenting words in a foreign language affects associative memory in young and older adults.

### 1.2 Influence of strategy use on the age-related associative deficit

Under which conditions strategy instructions may alleviate older adults’ episodic memory difficulties has been under debate for decades, with still no consensus. Older adults are thus less likely than young adults to spontaneously use effective memory strategies (Craik and Lockhart, 1972; Shing et al., 2009; Craik and Rose, 2012), which may be linked to age-related decline in the prefrontal cortex (PFC), which plays a key role in executive function (Diamond, 2012) and to a lack of cognitive resources (Hasher and Zacks, 1979; Salthouse, 1990). Several studies have reported that effective strategy instruction or training (such as generating interactive mental images or sentences that integrate the to-be-encoded stimuli) can help older adults reduce their age-related associative deficit (Bastin et al., 2013; Dulas and Duarte, 2013; Endemann and Kamp, 2022; Naveh-Benjamin et al., 2007). However, other studies have reported that older adults did not benefit from strategy instructions (Kamp et al., 2018) or benefitted to a lesser degree than young adults (Bridger et al., 2017), depending on characteristics of the strategies like the extent to which they depend on prior knowledge.

One promising type of strategy that may be especially effective for supporting associative memory in older adults is unitization (Graf and Schacter, 1989)—the integration of different information units into a single, holistic representation, such that the application enables older adults to rely on their intact item memory to retrieve the association (Ahmad et al., 2015; Bastin et al., 2013). One unitization strategy that has yielded promising results is, when concrete stimuli (objects or words) are to be encoded together with a specific color (presented in the background), the instruction to imagine the object in the paired specific color. Younger (Diana et al., 2008) and healthy older adults (Bastin et al., 2013), as well as patients with memory impairments (Diana et al., 2009), show improved associative performance with such instructions. However, the circumstances under which this may be an effective strategy to reduce older adults’ associative memory deficit remains an open question. For instance, the nature of the study material to which the strategy is applied may play a role in whether or not older adults can use it to their advantage.

### 1.3 Role of cognitive reserve

In order to better understand the age-related associative memory deficit, it is important to replicate it in different kinds of samples; such as with different levels of cognitive vitality. There is a series of protective factors (e.g., education, occupational levels, leisure activities), which enhance the brain’s ability to compensate for severe age-related changes, known as “cognitive reserve” (CR; Stern, 2003). Those older adults with higher CR, for instance, are also those with better working memory and episodic memory performance than those with low CR (Borella et al., 2023; Schwarz et al., 2024) and make more effective use of memory strategies (Frankenmolen et al., 2018). Among CR proxies, foreign language acquisition plays a prominent role (Ware et al., 2021), as it can lead to improvements in inhibition, working memory, and attentional switching (Garbin et al., 2010; Ware et al., 2021). It also appears not only to improve recall accuracy, but also to enhance the ability to form conceptual associations between words and contexts (Francis et al., 2018; Van Hell and De Groot, 1998) and leads to functional and structural changes, particularly in fronto-parietal networks and the hippocampus, which are associated with memory and executive control (Bubbico et al., 2019; Mårtensson and Lövdén, 2011). Whitford and Titone (2016) reported that older bilingual adults have slower global reading speeds, suggesting that lifelong or later foreign language learning may train visual attention strategies that can compensate for age-related decline. Higher CR can thus strengthen cognitive control processes important for associative binding.

### 1.4 The present study

In the present study, we attempted to replicate the associative memory deficit in younger and older foreign language learners, who are expected to show higher CR than people who do not study a foreign language. We captured item and associative memory using a word–color association task in both their native (Italian) and a second language which they were learning (German). If older adults prioritize item over associative encoding, then the increased item-processing demands of the foreign-language condition should amplify their age-related associative memory deficit. Secondly, we examined whether older adults benefit from a unitization strategy instruction, which required participants to form an integrated mental image of the word’s meaning (e.g., an apple) in the presented background color (e.g., yellow), thereby promoting the incorporation of contextual information into an item-level memory trace. We further hypothesized the unitization strategy to be more readily applicable in the native language, yielding a greater performance benefit in that condition than in the foreign language condition.

## 2 Methods

This study was reviewed and approved by the local ethics committees at the University of Padua and Trier University before data collection. All participants provided their written informed consent.

### 2.1 Participants

Italian native speakers were recruited through German language courses in the areas of Milan, Bologna, and Padua; and through the ERASMUS exchange student program at Trier University. To be included in the study, participants had to have a minimum language proficiency level of A2 in German, with an upper limit of B2 (self-report), and be 18–35 (young adults) or at least 60 years (older adults) old. Color blindness was an exclusion criterion. Participants did not receive a monetary compensation.

The sample size was originally planned to detect an interaction in an age group (young vs. old) x memory type (item vs. associative memory) mixed factors ANOVA with a medium effect size of *f* = 0.25, *α* = 0.05, and 1-*β* = 0.8. A minimum of N = 34 (*n* = 17 participants per group), were required (Faul et al., 2007). We thus collected as many datasets as possible in a fixed time period, with the goal of collecting at least 17 datasets per group.

Thirty-four young (age: M = 22.5; SD = 3.54; 11 male/23 female) and 17 older adults (M = 68.59; SD = 5.24; 5 male/12 female) participated. The older adults completed the Mini-Mental State Examination (MMSE; Folstein et al., 1975), and no participant demonstrated indications of cognitive impairment (all scores >28 at the MMSE; Table 1).

**Table 1:** Demographic information and dependent variables (mean +/− SD).

Measure	Young (all)	Young High-K	Young Low-K	Old
*N*	34	17	17	17
Demographics				
Age	22.50 (3.60)	22.94 (3.44)	22.06 (3.80)	68.59 (5.60)
Sex: %M	32	24	41	29
%F	68	76	59	71
Level: A2%	9	6	12	-
B1%	50	41	59	76
B2%	41	53	29	24
Questionnaires				
BDI-II	11.47 (7.35)	10.76 (8.33)	12.18 (6.39)	-
GDS	-	-	-	1.59 (1.28)
VVIQ	2.07 (0.75)	2.23 (0.91)	1.90 (0.52)	1.84 (0.66)
CR_c*	52.27 (9.76)^1^	54.29 (11.42)	50.13 (7.38)^1^	58.35 (7.25)
CR_r	-	-	-	31.53 (6.97)
Vocabulary test				
% correct translation**	47.35 (17.08)	55.29 (17.61)	39.41 (12.56)	62.88 (15.09)
% Tip of the Tongue	17.61 (19.88)	26.54 (23.91)	8.69 (8.62)	13.20 (8.83)
% “known” (Correct translation + ToT)*	64.97 (20.28)	81.83 (12.15)	48.10 (9.81)	76.08 (15.68)
% color recall**	41.01 (17.06)	46.67 (17.48)	35.36 (15.06)	24.58 (18.84)
Encoding judgment				
*Proportion of judgment of “very well” or “rather well” in Blocks 3 + 4*				
Italian*	0.79 (0.15)	0.81 (0.15)	0.76 (0.17)	0.89 (0.11)
German	0.62 (0.13)	0.68 (0.10)	0.56 (0.12)	0.68 (0.14)
Memory performance				
*Blocks 1 + 2: No strategy instruction*				
Item PR *Italian*	0.72 (0.22)	0.75 (0.23)	0.70 (0.21)	0.60 (0.21)
Associative PR *Italian*	0.53 (0.30)	0.60 (0.28)	0.47 (0.32)	0.43 (0.28)
Item PR *German*	0.69 (0.20)	0.74 (0.18)	0.64 (0.21)	0.73 (0.14)
Associative PR *German*	0.49 (0.24)	0.47 (0.29)	0.52 (0.19)	0.35 (0.21)
*Blocks 3 + 4: Strategy instruction*				
Item PR *Italian*	0.87 (0.18)	0.88 (0.18)	0.86 (0.18)	0.82 (0.13)
Associative PR *Italian*	0.82 (0.16)	0.87 (0.12)	0.77 (0.19)	0.57 (0.28)
Item PR *German*	0.82 (0.13)	0.83 (0.15)	0.82 (0.12)	0.78 (0.14)
Associative PR *German*	0.54 (0.30)	0.63 (0.26)	0.44 (0.32)	0.44 (0.27)

** p < 0.01; *p < 0.05 (young vs. older adults); ^1^ One missing value.

### 2.2 Procedure

Participants were tested individually in a quiet room at the institution where they had been recruited. The entire experimental session was led by a trained experimenter. After signing the written informed consent, the older subjects completed the MMSE. All participants then filled out a demographic questionnaire. Next, all participants completed the item and associative memory task on a laptop, followed by a paper-based vocabulary test. At the end of the session, participants completed a questionnaire capturing depressive symptoms. The total duration of an experimental session was about 1.5 h.

### 2.3 Item and associative memory task

#### 2.3.1 Stimulus selection

A rating study was conducted to select stimuli for the experiment. Thus, 220 words were selected from the WWN database (Lahl et al., 2009). In an online questionnaire, 20 young Italians and 20 young Germans rated each word of a subset of 110 words individually in a number of dimensions. First, the word was shown in both Italian and German and the participants were asked to rate whether the words were phonetically and orthographically similar to each other (for example: “cielo” is dissimilar from “Himmel” (sky); “lampada” is similar to “Lampe” (lamp)) on a scale of 1 “very similar,” 2 “similar,” 3 “dissimilar” and 4 “very dissimilar.” This rating was obtained to preclude that presentation of an unknown foreign word would lead to its identification due to similarity to the same word in the native language. Then, the word was shown in the native language and was rated based on visual imageability on a scale of 1 “very well,” 2 “well,” 3 “okay,” 4 “poor” and 5 “very poor.” Finally, the word was shown in front of a blue, green, red and yellow background one after the other and participants rated how well they could create a mental image of the object in each respective color on a scale of 1 “very well,” 2 “well,” 3 “okay,” 4 “poor” and 5 “very poor.”

We selected words that were rated as “dissimilar” or “very dissimilar” by more than 50% of the participants; however, to reach the required number of stimuli, we additionally included three words that fell below the 50% threshold (mirror: “specchio” – “Spiegel”; bathroom: “bagno” – “Bad”; wheel: “ruota” – “Rad”; see Supplementary material). The general visualizability was rated as “very well” or “well” by between 70 and 100% of the sample. Each selected word was assigned two colors for which between 52 and 90% of the participants had rated the pair as relatively easy to imagine (ratings 1, 2 or 3) in order to be used during study and potentially as recombined pair during test. The two selected colors differed by no more than 12.5% in word-color imageability to minimize the reliance on factors other than associative memory per se during the recognition phase for recombined pairs.

Based on these criteria, we selected a total of 180 words, which were randomly divided into two sets of 90 distinct German and 90 distinct Italian words, respectively, each with 2 associated colors (selected from blue, green, red and yellow).

#### 2.3.2 Task and trial structure

**Figure 1:**
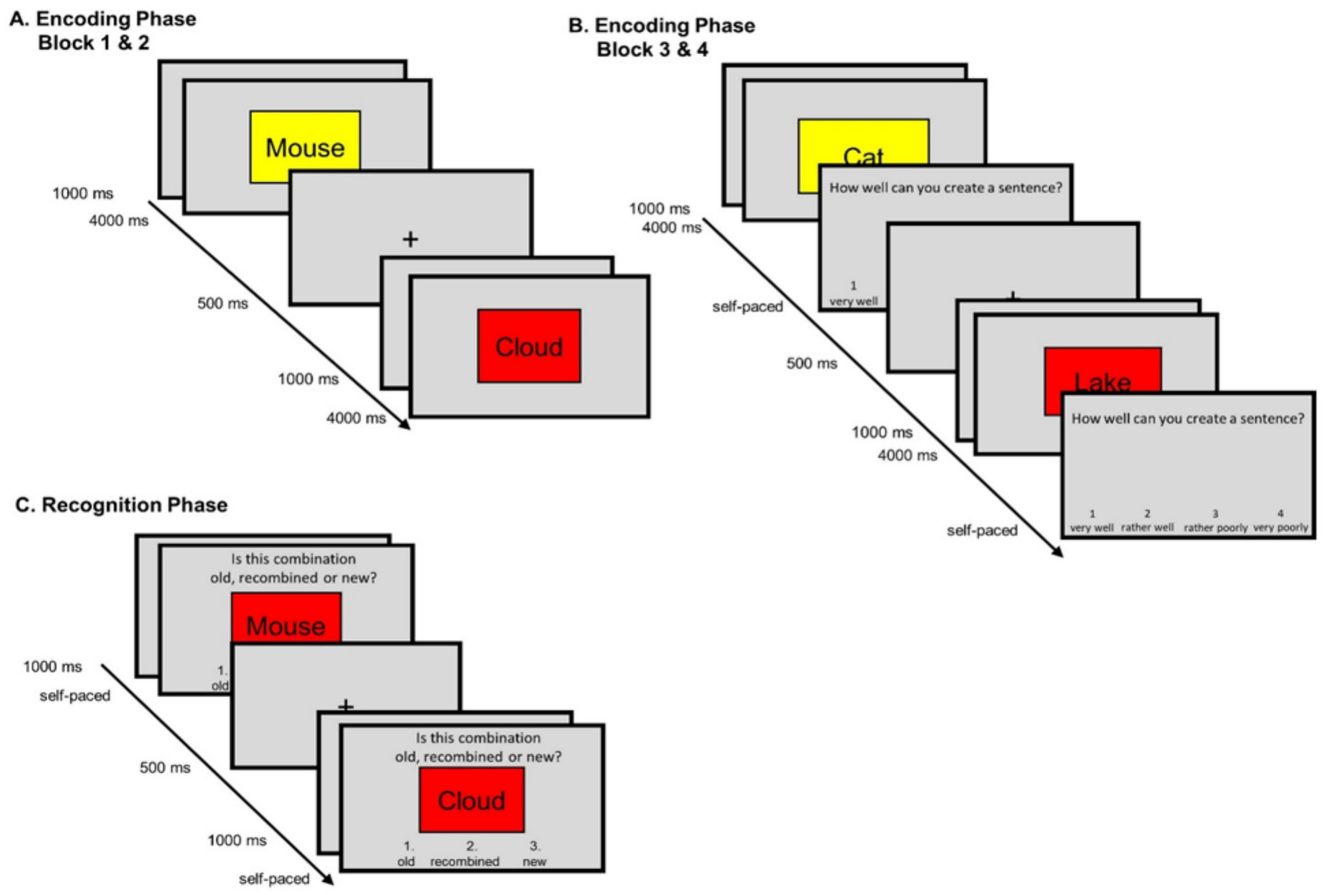
Trial structure from the encoding phase for blocks 1 and 2 (without a provided strategy; **A**) and for blocks 3 and 4 (with the instruction to visualize the object in the color and create a sentence; **B**). The recognition phase (**C**) is the same for all blocks; shown is one “old” trial (Cloud – red) and one “recombined” trial (Mouse – red).

The task was implemented using E-Prime 3.0 (Psychology Software Tools) and included 4 experimental blocks. Within a given block, all words were presented in one of the languages. The order of the blocks was either German-Italian-German-Italian (“German first”) or Italian-German-Italian-German (“Italian first”) and was determined randomly for each participant. Each word was presented in black Arial font in the middle of the screen in front of a colored square on a gray background (Figure 1).

Each block started with an encoding phase, in which 30 word-color pairs were presented sequentially. Each encoding trial started with a fixation cross displayed for 500 ms, followed by a blank screen of 1,000 ms, and the presentation of a word-color pair for 4,000 ms (Figure 1).

In blocks 1 and 2, participants were instructed to memorize 30 word-color pairs without being given specific mnemonic strategies.

In blocks 3 and 4, participants were instructed to visualize the object in the corresponding color and to mentally create a sentence accordingly (for example for phone + blue: “Luisa buys herself a new blue phone”). If a German (i.e., foreign) word was unknown, they were asked to visualize the written word in a font of the presented color instead. Following the word-color pair, the question “How well can you form a sentence?” was displayed with a scale from 1 (very well) to 4 (very poorly) shown below and participants provided their judgment in a self-paced manner using the laptop keyboard (Figure 1).

Following the encoding phase and in between two blocks, participants completed questionnaires for 2 min (The Vividness of Visual Imagery Questionnaire; VVIQ; Marks, 1973; and the Current and Retrospective Cognitive Reserve survey; 2CR survey; Borella et al., 2023). The recognition phase then commenced, in which 15 pairs were presented identically to those from the encoding phase (“old”), 15 consisted of previously studied words arranged with a new color (“recombined”), and 15 were entirely new (“new”). Each trial began with a fixation-cross for 500 ms and a blank screen for 1,000 ms, followed by the presentation of a word-color pair together with the rating scale of “old” (1), “recombined” (2), or “new” (3). The participant’s response terminated the screen (Figure 1).

During both encoding and retrieval, participants took a break after every 15th trial. The composition of the word-color combinations was counterbalanced, ensuring that each word was presented in Italian to one subject and in German to another, and which of the two associated colors was paired with each word was counterbalanced.

#### 2.3.3 Vocabulary test

Following completion of the experimental task, a paper-based vocabulary test was administered. Participants were presented with the complete list of German words previously shown in the memory task (*n* = 90) and were asked to provide the corresponding Italian translation. If they could not retrieve the translation but recognized the word, they could mark a box indicating a “tip of the tongue” (ToT) experience. If they did not know the word, they were instructed to leave the item unanswered.

Furthermore, participants were asked to indicate the background color with which each word had originally been presented to calculate recall accuracy of the correct color associated with each item. In cases where the word appeared in a recombined form during the recognition test, a response was considered correct if the participant accurately recalled the color presented at study or at test. At the very end of the session, the Beck Depression Inventory–II (BDI-II; Beck et al., 1998) was administered for young adults, while the Geriatric Depression Scale-Short version (GDS-15; Sheikh and Yesavage, 1986) was administered for older adults.

### 2.4 Questionnaires

The VVIQ (Marks, 1973) is a widely used self-report measure assessing visual mental imagery ability. Participants rate the clarity and vividness of their mental images across various scenarios on a 5-point Likert scale. Higher scores, computed as total divided by count, indicate greater imagery vividness.

The 2CR survey (Borella et al., 2023), is a comprehensive tool designed to assess CR across different life stages. It evaluates five dimensions: socioeconomic status, family engagement, leisure activities, social engagement, and spiritual or religious practices. The survey distinguishes between current status (CR_c, at the time of assessment) and retrospective status (CR_r, as recalled from young adulthood) periods, allowing for a nuanced understanding of how various life experiences contribute to CR over time. For young adults, only CR_c, while for older adults, both were captured.

The Geriatric Depression Scale – Short Version (GDS-15; Sheikh and Yesavage, 1986) and the Beck Depression Inventory-II (BDI-II; Beck et al., 1998) are self-report measures for assessing depressive symptoms specifically in older adults and generally in adults, respectively. Comprising 15 and 21 items, respectively, both the GDS-15 and the BDI-II result in a total score, with higher scores indicating stronger depressive symptoms.

### 2.5 Statistical analysis

The data were analyzed using SPSS 27 and JASP (JASP Team, 2026) (version 0.95.4) software. Questionnaire scores were compared between the groups with t-tests for independent samples.

To assess item and associative memory performance, corrected hit rates (Pr-scores) were computed by subtracting false alarm rates from hit rates, following Snodgrass and Corwin (1988). For the item score, responses of “old” or “recombined” to old and recombined pairs were classified as hits, whereas the same responses to new pairs were categorized as false alarms. For the associative score, “old” responses to old pairs were considered hits, while “old” responses to recombined pairs were designated as false alarms.

Based on their scores on the vocabulary test following the memory task, the group of young adults was sub-divided into a group with high vocabulary knowledge (High-K) and a group with lower vocabulary knowledge (Low-K), so that the key comparison reported here contains three groups (High-K, Low-K, old; see results section for further detail).

Pr-scores were analyzed using linear mixed models (LMMs) in JASP. The model contained the fixed effects group (High-K, Low-K, old), language (native/Italian, foreign/German), strategy (no instruction, strategy instruction), and memory type (item, associative), and all interactions, as well as the random effect participant, for which random intercepts were modeled. We initially generated a model additionally containing the fixed effect block order (Italian first, German first), but since its inclusion did not modulate the result pattern of the key variables, we chose the simpler model not containing order. Significant group effects and interaction effects were resolved by Holm-corrected sets of contrasts (with alpha = 0.05), in which the older adult group was contrasted with both groups of young adults and/or with the two groups (High-K, Low-K) separately, depending on hypothesis-relevance, as specified in the results section. All models used Type II sums of squares, and likelihood ratio tests to test for significance.

The participants’ judgments during the encoding task after strategy instructions were analyzed in a separate LMM.

## 3 Results

### 3.1 Sample characteristics and questionnaires

Demographic data and scores from standardized questionnaires and tests are shown in Table 1. Group differences between younger and older adults in the questionnaires were found in the CR_c, *t*(48) = 2.26, *p* = 0.028, *d* = 0.68, but not in any other questionnaire (all *p*-values > 0.05). This reflected a larger extent of current CR factors in older adults. All participants reported speaking at least one language other than Italian and German. Young adults reported a higher number of spoken languages (*M* = 2.03) than older adults (*M* = 1.35), *U* = 153, *z* = 2.93, *p* = 0.003.

### 3.2 Vocabulary test

Independent samples t-tests suggested that older adults had a higher percentage of correct translations than young adults, *t*(49) = 3.18, *p* = 0.003, *d* = 0.93 (Table 2). The age groups showed no significant differences in ToT, *t*(49) = 0.87, *p* = 0.39 (Table 1).

**Table 2:** Linear mixed model for recognition performance (Pr).

Fixed effect	df	χ^2^	*p*
Language	**1**	**16.478**	**< 0.001**
Memory Type	**1**	**105.928**	**< 0.001**
Strategy Instruction	**1**	**50.334**	**< 0.001**
Group	**2**	**7.869**	**0.020**
Language x Memory Type	**1**	**11.400**	**< 0.001**
Language x Strategy Instruction	**1**	**11.652**	**< 0.001**
Memory Type x Strategy Instruction	1	0.080	0.778
Language x Group	2	3.387	0.184
Memory Type x Group	**2**	**8.209**	**0.017**
Strategy Instruction x Group	2	0.668	0.716
Language x Memory Type x Strategy Instruction	1	2.436	0.119
Language x Memory Type x Group	2	0.608	0.738
Language x Strategy Instruction x Group	2	1.559	0.459
Memory Type x Strategy Instruction x Group	2	4.416	0.110
Language x Memory Type x Strategy Instruction x Group	**2**	**8.558**	**0.014**

The LMM used type 2 sums of squares and likelihood ratio tests. The LMM includes participant as a random effect; with modeled random intercepts. Bold faced values index statistically significant effects.

Due to the age difference in vocabulary test performance, and since twice as many datasets from young adults were available, we divided the young adult group into participants with high vocabulary knowledge (High-K) versus those with low vocabulary knowledge (Low-K) based on a median split for the sum of correct translations and ToT responses (median = 61.67%).

A comparison between the young High-K and Low-K groups yielded no significant differences in the questionnaire scores or in the number of other spoken languages (all *p-*values > 0.05; Table 1).

### 3.3 Memory performance (Pr-scores)

Significance tests of the LMM with the fixed factors group (young High-K vs. young Low-K vs. old), memory type (item vs. associative), language (native/Italian vs. foreign/German), and strategy instruction (none vs. interactive imagery), as well as participant as a random effect, are shown in Table 2. Post-hoc contrasts are shown in the Supplementary materials.

Significant effects of language, memory type and strategy instruction reflected higher performance in the native/Italian language, for item memory, and after strategy instruction.

Overall memory performance was highest in the High-K (*M* = 0.72, CI[0.66, 0.78]), followed by the Low-K (*M* = 0.66, CI[0.59, 0.71]) and the older adult group (*M =* 0.59, CI[0.53, 0.65]). Holm-corrected follow-up contrasts revealed significant differences between the young (High-K + Low-K) and older adults, which in pairwise comparisons was significant between the High-K and the older adult group (Supplementary Table 2.1).

The memory type x group interaction (Table 2) reflected a larger age difference (High-K + Low-K vs. older adults) in associative memory compared to item memory, replicating the age-related associative memory deficit. This pattern remained significant in the interaction contrast between High-K vs. older adults (Supplementary Table 2.2).

The memory type x group x strategy instruction interaction indicated that the age-related associative deficit increased from before to after the strategy instruction. In fact, without strategy instruction, the associative deficit was not significant overall, while afterward it was significant (Supplementary Table 2.3). Pairwise group contrasts of the interaction suggested that this pattern was also significant in comparison only to the High-K group (Supplementary Table 2.3).

Most importantly, the group x strategy x language x memory type interaction suggested that the age-related associative deficit was modulated by language and strategy instruction. Overall, the associative deficit (group x memory type interaction) was trend-level significant only for the foreign/German language without strategy instruction, but significant only in Italian after strategy instruction (Supplementary Table 2.4). When comparing the older adults only to the High-K group, which was matched in vocabulary knowledge, the associative deficit was not significant in any language without the strategy instruction, but was significant for the native/Italian language after the strategy instruction (Supplementary Table 2.4).

A language x memory type interaction indicated that performance differences between the languages were observed for associative, but not item memory (Supplementary Table 2.5). The significant language x strategy instruction interaction indicated that the strategy instruction was more effective in the native/Italian language: While there was no significant difference in memory performance between the language conditions without the strategy instruction, performance was higher in the native/Italian than in the foreign/German condition after the strategy instruction (Supplementary Table 2.6).

### 3.4 Encoding judgment

The probability with which participants reported to be able to generate sentences of the words interacting with the background color “well” or “rather well” during the encoding phase in block 3 + 4, was analyzed in a LMM with the fixed effects group (High-K, Low-K, old) and language (native/Italian, foreign/German), as well as the random effect participant. All fixed effects were significant, group: χ^2^ (2, N = 51) = 13.08, *p* = 0.001, language: χ^2^ (1, N = 51) = 117.91, *p* < 0.001, group x language interaction: χ^2^ (1, N = 51) = 8.03, *p* = 0.018. In the native/Italian language, older adults provided higher encoding judgments (*M* = 0.89, CI[0.84, 0.95]) than both groups of young adults, with no significant difference between the two (High-K: *M* = 0.81, CI[0.76, 0.86]; Low-K: *M* = 0.76, CI[0.71, 0.84]). In the foreign/German language, the encoding judgment did not differ between the High-K (*M* = 0.68, CI[0.63, 0.74]), and the older adults (*M* = 0.68, CI[0.63, 0.73]), but was lower in the Low-K group (*M* = 0.56, CI[0.51, 0.61]).

## 4 Discussion

The present study investigated whether the age-related associative memory deficit in older adults is increased by stimulus presentation in a foreign language (German) compared to the native language (Italian) and examined whether a unitization strategy supports associative word-color linking in older adults in the two languages, thus alleviating the age-related associative memory deficit characterizing older adults. The insights gained regarding both main research questions are next discussed in turn.

### 4.1 Age-related associative memory deficit: modulation through foreign language?

Overall, we replicated the associative memory deficit in our sample of older adults, which consisted exclusively of foreign language learners, who showed relatively high CR_c scores, compared to prior studies (Feraco et al., 2024): The older adults’ associative memory was generally more strongly reduced, compared to the young adults, than their item memory. This effect was still obtained in a comparison only to the young adults with matched vocabulary knowledge (young High-K). This suggests that the age-related associative memory deficit is a phenomenon that is generally observed in different kinds of older adult samples, even those with particularly high CR.

However, unexpectedly, we found no evidence for an age-related associative deficit in older adults in the native language under intentional encoding conditions with no specific strategy instruction: Circumstances where an age-related associative deficit would be strongly expected based on prior literature (Old and Naveh-Benjamin, 2008). Possible reasons could concern our task design: Factors like a low processing difficulty of the stimulus pairs, the rate of presentation or the study list length, could have led to a relatively low overall task difficulty. However, associative memory performance was not near ceiling, speaking against this idea (Table 1).

**Figure 2:**
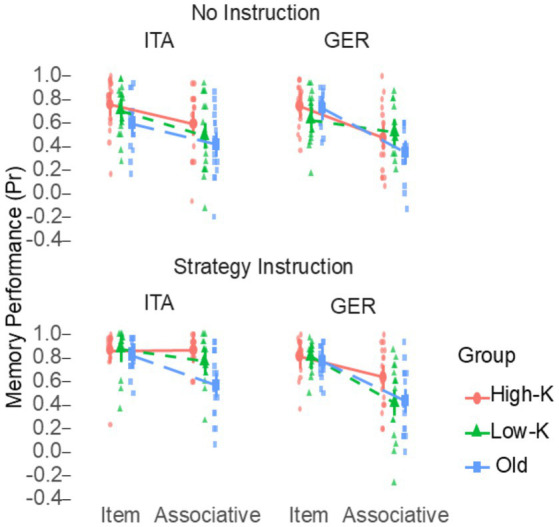
Memory performance (PR = hits rate-false alarm rate) by group (older adults, young adults with high vocabulary scores/High-K, young adults with low vocabulary scores/Low-K), memory type (item vs. associative), language and strategy instruction (no particular strategy instruction/ “learn” vs. unitization/ “sentence”).

Since CR in general (Stern, 2003) and foreign language learning in specific (Grossmann et al., 2021) is associated with reduced age-related neural and cognitive decline, it is possible that older adults with high CR do not show an associative deficit (yet), because they are able to compensate for associative memory difficulties in a task with no particular challenges (e.g., Peterson et al., 2017). That is, an age-related associative deficit was observed under similar encoding conditions, when stimuli were presented in the foreign language, and after strategy instruction (in the native language; blocks 3 and 4; Figure 2). When additional difficulties, like the presentation of stimuli in a foreign language or a challenging strategy, are introduced, older adults with high CR may no longer be able to compensate for memory difficulties.

The tendency for an increased associative memory deficit in the foreign language (without a strategy instruction) in principle supports our starting hypothesis that an enhanced item processing focus (here due to word presentation in a foreign language) may contribute to the relative deficit in associative memory in older adults. However, our *post hoc* contrasts cast doubt on this interpretation: The subgroup of young adults with matched vocabulary knowledge and CR_c—the High-K group—showed a performance pattern that was similar to the older adults, and in a targeted comparison between older adults and the High-K group there was no relative associative deficit in the foreign language either (without strategy instruction). This could imply that the tendency for enhanced item memory at the expense of reduced associative memory in the foreign language in the older adults in blocks 1 and 2 compared to the young adults as a whole group is due to the older adults’ higher foreign language proficiency or higher CR_c score at the group level in the present sample, compared to the whole present sample of young adults, rather than due to an age difference *per se*.

Thus, it has been suggested that professional competence with a foreign language affects the process of translating from one language to another. Dong and Chen (2025) demonstrated that professional translators employ more effective strategies in their translation processes, but at the same time allocate more resources to the implementation of these strategies in comparison to students with less language proficiency. Hence, it is possible that participants with relatively high foreign language proficiency (like our older adult and high-K sample) allocated more resources to processing the individual words in the foreign language. As a result, limited resources may remain for building associations, resulting in relatively reduced associative memory, and hence, in a trade-off between the effort in processing foreign language words and associations among them. However, this appears to be an age-invariant pattern and hence our results provide only very limited support for the notion that an enhanced item processing focus, at the expense of associative memory, contributes to the age-related associative memory deficit in older adults. Before the more general idea of item vs. associative processing tradeoffs contributing to age-related associative deficits is completely rejected, however, future studies should test this idea with meaningless stimuli, where older adults cannot rely on their relatively intact semantic processing (e.g., Mayr and Kliegl, 2000) to scaffold item processing.

### 4.2 Effectiveness of the unitization strategy in older adults

The strategy that participants were instructed to use in the present study—imagining the object in the presented color—was similar to a previously used unitization strategy. In this prior study, older adults were able to raise their associative memory performance to that of young adults by applying this strategy to encode object-color pairs in their native language (Bastin et al., 2013). Our results differ from these prior results: Strategy-related benefits in associative memory were less pronounced in older than in young adults, resulting in an increased relative associative memory deficit in older, versus young, adults after strategy instruction. This pattern was observed despite the fact that older adults were more likely to judge that they could generate a sentence “very well” or “rather well.”

Several differences between the present task and Bastin et al. (2013) may explain the difference in result pattern. First, in the present study, participants had to generate their own sentence, while in Bastin et al. (2013), a sentence was provided on-screen. One reason that we made this decision is because the method would be of much higher practical use if it was effective without external input on specific supporting sentences. Since the presented colors were not the typical color of the object and hence imaging the object in the color may have been incongruent with pre-existing semantic knowledge, it may have been much more challenging for the older adults to generate such an explanation and hence a unitized representation (see also Bridger et al., 2017; Endemann and Kamp, 2022; Kamp et al., 2018). Interestingly, Khoo and Tibon (2025) reported that in a different unitization task, the requirement to generate one’s own mediator (i.e., sentence to associate word pairs) reduced the beneficial effect of unitization (versus a control strategy) on associative memory across age groups. The role of self-generating sentences in paradigms like the present one is important to examine in further research.

Another difference is that Bastin et al. (2013)’s control condition required imagery of the object in interaction with a separate object of the given color. In our control condition, we allowed participants to encode the word-color pairings in any way they chose in order to provide a more natural and less constrained encoding context. Although it would appear that with the present control condition, there should be even more room for improving associative memory through the instructed strategy, in turn enhancing the chance to observe a beneficial strategy effect, the influence of this difference should be examined further.

Further differences are that Bastin et al. (2013) used only 2 different colors. In our study, each object was encoded with one of four colors, which we implemented to make the strategy more versatile and to reduce the proportion of objects associated with a given color. This could have potentially led to differences in task difficulty. We also implemented an associative recognition test in order to be able to estimate both item and associative memory, while Bastin et al. (2013) used a source memory test and reported source, but not item memory. A final important difference is that due our control condition not containing any particular encoding instructions, making carryover effects very likely if the control condition followed the strategy instruction blocks, we presented the different strategy conditions in a fixed order, with the no-strategy-instruction blocks always preceding the strategy blocks. We hence could not control for task-related training or fatigue effects.

In sum, the present results call for further research to examine under which conditions older adults benefit from mnemonic strategies in general, and from this kind of unitization strategy in specific. It should be noted that although the strategy-related performance benefit was larger in young adults, older adults did also benefit from the strategy instruction (Figure 2). Hence, our results suggest that in practice, older adults, at least those with a current high CR and foreign language knowledge, do seem to benefit from the application of the present strategy, just not to the extent that it would reduce their age-related performance deficit compared to young adults.

In the foreign language (German), associative memory generally benefited less from the strategy instruction than in the native language (Italian), but the effects were similar, statistically speaking, across age groups. Even in the young adults, the strong enhancement of associative memory due to the strategy instruction, which was observed in the native language, was much attenuated in the foreign language. The ratings provided during encoding also showed this pattern, as the proportion of trials in which participants reported to be able to generate a sentence “very well” or “rather well” was lower in the foreign language in all groups, and particularly so for the Low-K young adults. It hence appears that under more difficult processing conditions (i.e., in a foreign language), young adults showed similar difficulties in applying mnemonic strategies to older adults. This may be due to the fact that processing the individual words bound cognitive resources, leaving too little resources to effectively apply the strategy.

In sum, while under typical circumstances (i.e., in their native language), associative memory of young adults benefited more strongly from the strategy instruction than associative memory of older adults, thus amplifying the relative age-related associative deficit, in the foreign language, all groups benefited less from the strategy instruction. It would be interesting for future research to record reaction times during encoding to test whether the implementation of the strategy took more time in the foreign language. Further, it would be beneficial to record brain activity such as ERPs. Young adults typically show a frontal slow wave SME during associative encoding with effective memory strategies, presumably reflecting working-memory based elaboration (Kamp and Zimmer, 2015; Kamp et al., 2022; Mecklinger and Kamp, 2023). It would be, finally, interesting to examine whether this SME would be reduced when presenting items in a foreign language.

### 4.3 Limitations and conclusion

A major limitation to the present study is the small sample size, resulting from a difficulty in recruiting participants with these particular inclusion criteria (older adults participating in a foreign/German language class with a particular range of proficiency levels). Although we did reach the *a priori* minimum sample size in both age groups, the sample of older adults did not allow for additional analyses regarding individual differences. Further, we did not capture an exhaustive number of individual difference factors such as other proxies for CR like education.

In conclusion, older foreign language learners (a sample with relatively high current cognitive reserve factors) showed no disproportionate age-related deficits in associative memory under low-demand conditions (i.e., performing a memory task in their native language without using an instructed memory strategy). It would hence be worthwhile to further examine the potential of foreign language learning as interventions in older adults with and without cognitive decline, to build up cognitive reserve and the ability to compensate for cognitive challenges (e.g., Klimova and de Paula Nascimento e Silva, 2024; Vega-Mendoza et al., 2024).

However, in conditions with presumably increased cognitive demands, either through stimulus presentation in a foreign language or through the requirement to apply an instructed strategy, a relative age-related associative deficit emerged. This confirms a benefit of cognitive reserve in cognitive performance and suggests that CR could not compensate older adults’ low associative memory performance under the more difficult and resource consuming conditions.

Since the enhanced associative deficit of older adults in the foreign language on the group level seemed to be explained by their higher foreign language knowledge rather than representing a true age-related effect, we found little evidence for a contribution of an item processing focus to the age-related associative memory deficit.

The present study contributes to a better understanding of the boundary conditions of strategy-related benefits in older adults, especially those with a successful/ active aging process as shown by their current cognitive reserve level. Thus, young adults benefited more strongly from the unitization strategy, which presumably required a high level of self-initiated processing, effectively amplifying the age-related associative memory deficit in older adults. In the foreign language, associative memory of both young and older adults did not benefit from the instructed strategy, suggesting that processing stimuli in a foreign language leaves too little resources to effectively apply the strategy.

## Data Availability

The datasets presented in this study can be found in online repositories. The names of the repository/repositories and accession number(s) can be found at: https://osf.io/sd27h/.
