# Supplementary material for: The influence of foreign language and a word-color unitization strategy on the age-related associative deficit
Source: Front Psychol. 2026 Jul 28;17:1824417. doi: 10.3389/fpsyg.2026.1824417 (PMC13457631; doi:10.3389/fpsyg.2026.1824417)
Supplement: Supplementary file 1 [file Supplementary_file_1.DOCX]

Supplementary Material

***Overview***

1. **Stimuli**

This section lists all words used in the study in both Italian and German.

2. **Linear mixed model significance tests of fixed effects: Post Hoc contrasts**

Tables are provided that include the post hoc contrasts to follow up on significant main or interaction effects in the LMM (Table 2)

1. **Stimuli**
   1.1 **Italian**

FRECCIA, PIEDE, PAESE, CILIEGIA, UOVO, FARINA, ANATRA, RUOTA, MACCHIA, ESCAVATORE, RIGA, TRIFOGLIO, MUCCA, PONTE, PERA, BARCA, TRENO, NUVOLA, GUFO, TOPO, CERVELLO, SECCHIO, LUNA, CINTURA, DENTE, RIFUGIO, PRUGNA, POLTRONA, PALLONE, ANELLO, FANTASMA, RECINTO, OCCHIO, CUORE, CANNA, SEDIA, STANZA, MONTAGNA, PECORA, BOTTIGLIA, CIELO, ORECCHIO, VERDURA, SCIMMIA, BOCCA, TERRENO, LUMACA, ERBA, CERCHIO, GLOBO, CUCCHIAINO, CAVALLO, LATTE, RAMO, SIGNORE, FACCIA, LATTINA, PREZZEMOLO, FORCHETTA, PANCIA, TAVOLO, SAPONE, GESSO, MOLLA, UVETTA, PATATA, UCCELLO, CARAMELLA, CASA, ARANCIA, SPILLA, PIATTO, BAGNO, FUNGO, DENARO, CUSCINO, FRULLATORE, ALLUCE, FIUME, PORTO, TORTA, SERPENTE, FORNO, TESTA, TACCUINO, GELATO, CIPOLLA, LIBRO, GRANCHIO, SUCCO, CANE, SPECCHIO, FARFALLE, CANTINA, CHIESA, OROLOGIO, SCALA, COLLANA, NAVE, MACCHINA, COPERTA, ARMADIO, CONDOTTO, GATTO, MONDO, STOVIGLIE, COLTELLO, BICCHIERE, LETTERA, CAPELLO, ASINO, DITO, LETTO, BERRETTO, COLEOTTERO, SCIVOLO, GONNA, SCARPA, SPIAGGIA, CEROTTO, GALLO, CARTELLO, CITTÀ, CAMPANA, REMO, APE, TACCHINO, ONDA, PADELLA, SCAFFALE, CASCO, GRAFIA, GAMBA, CESTO, BISCOTTO, LINGUA, MANO, RAGNO, PIANOFORTE, FRUTTA, GALLERIA, PORTA, FAZZOLETTO, IMMAGINE, FULMINE, PANE, LAVAGNA, FIORE, FAGIOLO, ORSO, RANA, CHIAVE, VALIGIA, TETTO, GIORNALE, AGLIO, FORMAGGIO, PENTOLA, TAURO, LEPRE, CASTELLO, SASSO, LANA, CANDELA, REGALO, SPAZZOLA, CHIODO, PANTALONE, CAMICIA, NEVE, PROSCIUTTO, RISCALDAMENTO, PESCE, MIELE, BANDIERA, BOSCO, STRACCIO, LAGO, QUADERNO

1.2 **German**

PFEIL, FUSS, DORF, KIRSCHE, EI, MEHL, ENTE, RAD, FLECK, BAGGER, LINEAL, KLEE, KUH, BRÜCKE, BIRNE, BOOT, ZUG, WOLKE, EULE, MAUS, GEHIRN, EIMER, MOND, GÜRTEL, ZAHN, HÜTTE, PFLAUME, SESSEL, BALL, RING, GESPENST, ZAUN, AUGE, HERZ, ANGEL, STUHL, ZIMMER, BERG, SCHAF, FLASCHE, HIMMEL, OHR, GEMÜSE, AFFE, MUND, BODEN, SCHNECKE, GRAS, KREIS, KUGEL, LÖFFEL, PFERD, MILCH, AST, MANN, GESICHT, DOSE, PETERSILIE, GABEL, BAUCH, TISCH, SEIFE, KREIDE, FEDER, ROSINE, KARTOFFEL, VOGEL, BONBON, HAUS, APFELSINE, NADEL, TELLER, BAD, PILZ, GELD, KISSEN, MIXER, ZEH, FLUSS, HAFEN, KUCHEN, SCHLANGE, OFEN, KOPF, BLOCK, EIS, ZWIEBEL, BUCH, KRABBE, SAFT, HAI, HUND, SPIEGEL, SCHMETTERLING, KELLER, KIRCHE, UHR, LEITER, KETTE, SCHIFF, AUTO, DECKE, SCHRANK, ROHR, KATZE, ERDE, GESCHIRR, MESSER, GLAS, BRIEF, HAAR, ESEL, FINGER, BETT, MÜTZE, KÄFER, RUTSCHE, ROCK, SCHUH, STRAND, PFLASTER, HAHN, SCHILD, STADT, GLOCKE, RUDER, BIENE, PUTE, WELLE, PFANNE, REGAL, HELM, SCHRIFT, BEIN, KORB, KEKS, ZUNGE, HAND, SPINNE, KLAVIER, OBST, TUNNEL, TÜR, TUCH, BILD, BLITZ, BROT, TAFEL, BLUME, BOHNE, BÄR, FROSCH, SCHLÜSSEL, KOFFER, DACH, ZEITUNG, KNOBLAUCH, KÄSE, TOPF, STIER, HASE, SCHLOSS, STEIN, WOLLE, KERZE, GESCHENK, BÜRSTE, NAGEL, HOSE, BLUSE, SCHNEE, SCHINKEN, HEIZUNG, FISCH, HONIG, FAHNE, WALD, LAPPEN, SEE, HEFT

2. **Post hoc contrasts for significant fixed effects in the LMM on memory performance (Pr):**
   2.1. Group effect

|  | | | | | | | |
| --- | --- | --- | --- | --- | --- | --- | --- |
|  | | | | 95% CI | |  | |
|  | Estimate | SE | df | Lower | Upper | z | p† |
| Young (High-K+Low-K) vs. Old | 0.097 | 0.039 | ∞ | 0.021 | 0.174 | 2.499 | .037 |
| High-K vs. Old | 0.131 | 0.045 | ∞ | 0.043 | 0.219 | 2.916 | .014 |
| Low-K vs. Old | 0.063 | 0.045 | ∞ | -0.025 | 0.152 | 1.411 | .265 |
| High-K vs. Low-K | 0.068 | 0.045 | ∞ | -0.020 | 0.156 | 1.505 | .265 |
|  | | | | | | | |
| † P-values are adjusted using Holm adjustment. | | | | | | | |

2.2. Group x Memory Measure

|  | | | | 95% CI | |  | |
| --- | --- | --- | --- | --- | --- | --- | --- |
|  | Estimate | SE | df | Lower | Upper | z | p† |
| Young (High-K+Low-K) vs. Old | 0.104 | 0.038 | ∞ | 0.030 | 0.179 | 2.735 | .012 |
| High-K vs. Old | 0.129 | 0.044 | ∞ | 0.043 | 0.215 | 2.925 | .010 |
| Low-K vs. Old | 0.080 | 0.044 | ∞ | -0.006 | 0.166 | 1.812 | .070 |
|  | | | | | | | |

2.3. Memory Measure x Group x Strategy

|  | | | | | | | |
| --- | --- | --- | --- | --- | --- | --- | --- |
|  | | | | 95% CI | |  | |
|  | Estimate | SE | df | Lower | Upper | z | p† |
| No Strategy:  Young (High-K+Low-K) vs. Old x Memory Measure | 0.079 | 0.054 | ∞ | -0.027 | 0.185 | 1.460 | .144 |
| Strategy Instruction:  Young vs. Old x Memory Measure | 0.130 | 0.054 | ∞ | 0.024 | 0.236 | 2.408 | .032 |
|  | | | | | | | |

|  | | | | | | | |
| --- | --- | --- | --- | --- | --- | --- | --- |
|  | | | | 95% CI | |  | |
|  | Estimate | SE | df | Lower | Upper | z | p† |
| No Strategy:  High-K vs. Old x Memory Measure | 0.061 | 0.060 | ∞ | -0.056 | 0.177 | 1.020 | .616 |
| Strategy Instruction:  High-K vs. Old x Memory Measure | 0.205 | 0.060 | ∞ | 0.088 | 0.322 | 3.440 | .002 |
| No Strategy:  Low-K vs. Old x Memory Measure | 0.115 | 0.060 | ∞ | -0.004 | 0.233 | 1.894 | .175 |
| Strategy Instruction:  Low-K vs. Old x Memory Measure | 0.043 | 0.060 | ∞ | -0.076 | 0.161 | 0.710 | .616 |
|  | | | | | | | |

2.4. Memory Measure x Group x Strategy x Language

|  | | | | | | | |
| --- | --- | --- | --- | --- | --- | --- | --- |
|  | | | | 95% CI | |  | |
|  | Estimate | SE | df | Lower | Upper | z | p† |
| No strategy, German:  Young (High-K+Low-K) vs. Old x Memory Measure | 0.180 | 0.076 | ∞ | 0.031 | 0.330 | 2.361 | .055 |
| With strategy, German:  Young vs. Old x Memory Measure | 0.058 | 0.076 | ∞ | -0.092 | 0.207 | 0.758 | .897 |
| No strategy, Italian:  Young vs. Old x Memory Measure | -0.023 | 0.076 | ∞ | -0.172 | 0.127 | -0.296 | .897 |
| With strategy, Italian:  Young vs. Old x Memory Measure | 0.202 | 0.076 | ∞ | 0.052 | 0.351 | 2.647 | .032 |
|  | | | | | | | |
|  | | | | | | | |

|  | | | | | | | |
| --- | --- | --- | --- | --- | --- | --- | --- |
|  | | | | 95% CI | |  | |
|  | Estimate | SE | df | Lower | Upper | z | p† |
| No strategy, German:  High-K vs. Old x Memory Measure | 0.104 | 0.088 | ∞ | -0.069 | 0.276 | 1.177 | .479 |
| With strategy, German:  High-K vs. Old x Memory Measure | 0.153 | 0.088 | ∞ | -0.020 | 0.326 | 1.736 | .248 |
| No strategy, Italian:  High-K vs. Old x Memory Measure | 0.014 | 0.088 | ∞ | -0.159 | 0.186 | 0.156 | .876 |
| With strategy, Italian:  High-K vs. Old x Memory Measure | 0.245 | 0.088 | ∞ | 0.072 | 0.418 | 2.782 | .022 |
|  | | | | | | | |
|  | | | | | | | |

2.5. Language x Memory Type

|  | | | | | | | |
| --- | --- | --- | --- | --- | --- | --- | --- |
|  | | | | 95% CI | |  | |
|  | Estimate | SE | df | Lower | Upper | z | p† |
| Associative:  German vs. Italian | -0.142 | 0.025 | ∞ | -0.192 | -0.092 | -5.588 | < .001 |
| Item:  German vs. Italian | -0.017 | 0.025 | ∞ | -0.067 | 0.033 | -0.655 | .512 |
|  | | | | | | | |
|  | | | | | | | |

2.6. Language x Strategy

|  | | | | | | | |
| --- | --- | --- | --- | --- | --- | --- | --- |
|  | | | | 95% CI | |  | |
|  | Estimate | SE | df | Lower | Upper | z | p† |
| No Strategy  German vs. Italian | -0.016 | 0.025 | ∞ | -0.066 | 0.034 | -0.628 | .530 |
| With Strategy  German vs. Italian | -0.143 | 0.025 | ∞ | -0.193 | -0.093 | -5.616 | < .001 |
|  | | | | | | | |
|  | | | | | | | |
